# The technique of bladder implantation: further results and an assessment.

**DOI:** 10.1038/bjc.1968.96

**Published:** 1968-12

**Authors:** D. B. Clayson, J. A. Pringle, G. M. Bonser, M. Wood


### THE TECHNIQUE OF BLADDER IMPLANTATION: FURTHER RESULTS AND AN ASSESSMENT

D. B. CLAYSON, J. A. S. PRINGLE, G. M. BONSER AND M. WOOD

*From the Department of Experimental Pathology and Cancer Research, The School of Medicine, Leeds, 2*

Received for publication August 8, 1968

JULL (1951) suggested that the surgical introduction of a pellet containing a test chemical into the lumen of the mouse bladder might be useful for routine testing for carcinogenic activity. The method, it was thought, would possess the following advantages: (i) the chemical would be slowly eluted from the pellet and would therefore remain in contact with the bladder epithelium for a prolonged period; (ii) the metabolic processes of the liver, etc., would be by-passed, and (iii) the bladder would function under approximately normal conditions.

Bladder implantation has been used successfully in Leeds (Bonser, Clayson and Jull, 1958, 1963), in London (Allen, Boyland, Dukes, Horning and Watson, 1957) and in Madison (Bryan, Brown and Price, 1964*a*, *b*).

The advantages predicted for the technique have not been completely fulfilled. Chemicals have been shown to diffuse from pellets at different rates (Bryan, Brown, Morris and Price, 1964), although there was no correlation between rate of diffusion and carcinogenicity of a series of chemicals. The bladder epithelium has been shown to be permeable to certain chemicals (Bryan, Morris and Brown, 1965; Pringle, 1966), and so it cannot be assumed that metabolism by the liver with consequent excretion of metabolites is necessarily excluded by the use of bladder implantation. Furthermore, the presence of a foreign body, the pellet, in the bladder lumen affects the response of the epithelium to a carcinogen (Bryan and Springberg, 1966) probably because it induces mitosis in the bladder epithelium (Clayson and Pringle, 1966). The pellet by itself usually leads to a background incidence of tumours (Bonser *et al.*, 1958).

The purpose of this paper is to present new information on the testing of chemicals by bladder implantation and to reassess the utility of the method. The chemicals investigated consist of aromatic amines and their derivatives, hydrocarbons and dyestuffs.

### MATERIALS AND METHODS

*Animals*.—C57 × IF $F_1$ hybrid mice were bred in the laboratory and maintained on Oxo Diet 41B and water, *ad libitum*. They were 10–12 weeks of age at the start of the experiment.

Bladder implantation was carried out by the method of Jull (1951) as modified by Allen *et al.* (1957). Except where otherwise stated, the experiments were terminated at 40 weeks and the bladders prepared for histology in the usual way. The histological grading of the tumours was assessed by the criteria of Bonser and Jull (1956).

*Chemicals*.—1-Methoxy- and 3-methoxy-2-acetamidofluorene were a gift from Dr. H. R. Gutmann (Minneapolis, U.S.A.); Blue VRS and Patent Blue V were a gift from Dr. A. Munn (Imperial Chemical Industries Ltd.); Oil Orange KB was from a sample presented to us, some 10 years ago, by Messrs. Pointing of Hexham. 1,2-Benzanthracene and 20-methylcholanthrene were purchased from Koch-Light, Ltd., Colnbrook, Bucks.

1-Phenylazo-2-naphthol was a chromatographically purified sample purchased from The British Drug Houses, Ltd., Poole, Dorset. 1,2-Naphthoquinone-4-sulphonic acid (sodium salt) and *o*-aminoazotoluene were purchased from the same source and used without further purification. N-Hydroxy-2-acetamido-fluorene, N-hydroxy-4-acetamidodiphenyl and 2,5-dimethoxyphenylazo-2-naphthol were synthesised by standard procedures in the laboratory.

Chemicals were mixed with enough crushed paraffin wax to make a 12·5% suspension and compressed into pellets weighing 15–17 mg. Glass beads were of 4 mm. diameter and weighed approximately 100 mg.

### RESULTS AND COMMENT

The survival of the implanted animals in these experiments was similar to that found previously. Of 1429 mice, 1177 (82·4 per cent) survived to 40 weeks of age. The mortality was randomly distributed among the different groups.

Two samples of paraffin wax were used. The first was the sample of crushed paraffin wax described by Bonser, Boyland, Busby, Clayson, Grover and Jull (1963) and, when implanted alone, gave tumour yields of 4·5 and 3·8 per cent in two experiments (Table I). A further batch of paraffin wax was used after the previous sample was exhausted. This proved to be more carcinogenic causing 8·7 per cent of tumours in female mice but none in an equivalent group of male mice (Table II).

The highest yield of tumours was obtained with a commercial sample of Oil Orange KB, which is made by coupling diazotised *p*-xylidine with 2-naphthol. In the first experiment, this induced carcinomas of the bladder epithelium in all but one of the 44 surviving animals. These carcinomas were more advanced than usual, 9 having spread to structures outside the bladder (Table I). In a second experiment the sample of this chemical implanted in the new batch of paraffin wax induced bladder carcinomas in 40 of 62 surviving mice but these tumours were less advanced than in the first test (Table II). From the order in which the different chemicals had been implanted, it was considered possible that the pellets in the first experiment might have been contaminated with traces of a carcino-genic polycyclic hydrocarbon. It was therefore decided to examine the effect of small amounts of 20-methylcholanthrene on the carcinogenicity of 1-phenyl-azo-2-naphthol. Groups of mice were set up with paraffin wax alone, with 0·5 per cent 20-methylcholanthrene, with 12·5 per cent 1-phenylazo-2-naphthol and with both chemicals together. 20-Methylcholanthrene in this concentration caused 11 per cent and 1-phenylazo-2-naphthol 23 per cent of carcinomas. In combination the chemicals induced 43 per cent carcinomas of which 3 (11 per cent) had penetrated the bladder wall. This experiment shows that the hydrocarbon and azo compound together produced more numerous, and more advanced, tumours than either alone. Whether or not there was hydrocarbon contamina-

TABLE I.—*Bladder Implantation Experiments using Crushed Paraffin Wax as a Vehicle*

| Chemical added | Number of mice at start | Effective number of mice | Number with concretion | Number with squamous metaplasia | Number with tumours† Papilloma | Carcinoma I | Carcinoma II | Carcinoma III | Total | Per cent | $X^2$‡ | P‡ |
|---|---|---|---|---|---|---|---|---|---|---|---|---|
| None | 106 | 89 | 0 | 1 | 1 | 3 | 1 | 0 | 4 | 4·5 | — | — |
| None* | 70 | 53 | 0 | 0 | 0 | 2 | 0 | 0 | 2 | 3·8 | — | — |
| *Amines and Derivatives* | | | | | | | | | | | | |
| o-Aminoazotoluene | 60 | 52 | 0 | 2 | 0 | 7 | 2 | 0 | 9 | 17·3 | 9·131 | <0·01 |
| N-Hydroxy-2-acetamidofluorene | 86 | 72 | 0 | 3 | 1 | 4 | 1 | 0 | 5 | 6·9 | 0·724 | >0·05 |
| N-Hydroxy-4-acetamidodiphenyl | 93 | 82 | 6 | 3 | 1 | 5 | 3 | 0 | 8 | 9·8 | 2·714 | >0·05 |
| 1, 2-Naphthoquinone-4-sulphonic acid (sodium salt) | 54 | 48 | 0 | 1 | 0 | 4 | 1 | 0 | 5 | 10·4 | 2·488 | >0·05 |
| *Dyestuffs* | | | | | | | | | | | | |
| 1-Phenylazo-2-naphthol | 61 | 56 | 0 | 0 | 0 | 9 | 4 | 0 | 13 | 23·2 | 16·693 | <0·001 |
| 2, 5-Dimethoxy-1-phenylazo-2-naphthol | 53 | 50 | 0 | 2 | 0 | 5 | 2 | 0 | 7 | 14·0 | 7·002 | <0·01 |
| Orange KB | 60 | 44 | 0 | 11 | 0 | 7 | 27 | 9 | 43 | 97·7 | — | ≪0·001 |
| Blue VRS | 51 | 39 | 0 | 0 | 0 | 1 | 0 | 0 | 1 | 2·7 | — | 0·53§ |
| Patent Blue V | 45 | 39 | 0 | 1 | 0 | 0 | 2 | 0 | 2 | 5·1 | — | 0·76§ |
| *Hydrocarbons* | | | | | | | | | | | | |
| 20-Methylcholanthrene* (0·5 per cent) | 71 | 53 | 0 | 2 | 1 | 4 | 2 | 0 | 6 | 11·3 | 3·365 | >0·05 |
| 1-Phenylazo-2-naphthol + 20-Methylcholanthrene (0·5 per cent) | 74 | 62 | 1 | 4 | 1 | 22 | 2 | 3 | 27 | 43·5 | 49·214 | ≪0·001 |
| 1, 2-Benzanthracene | 77 | 52 | 0 | 2 | 1 | 11 | 6 | 0 | 17 | 32·7 | 29·327 | ≪0·001 |

* Quoted by Clayson and Pringle (1966).

† Most advanced lesion only.

‡ The two control groups were taken together.

§ The exact method for 2 × 2 tables was used.

TABLE II.—*Bladder Implantation in a Further Batch of Paraffin Wax*

| Chemical added | Number of mice at start | Effective number of mice | Sex | Number with concretion | Number with squamous metaplasia | Number with tumours* Papilloma | Carcinoma I | Carcinoma II | Carcinoma III | Total | Per cent | P‡ |
|---|---|---|---|---|---|---|---|---|---|---|---|---|
| None† . . . . | 53 | 46 | F | 0 | 0 | 0 | 4 | 0 | 0 | 4 | 8·7 | |
| None† . . . . | 61 | 56 | M | 0 | 0 | 0 | 0 | 0 | 0 | 0 | 0 | |
| *Amines* | | | | | | | | | | | | |
| 1-Methoxy-2-acetamido-fluorene . . . | 87 | 76 | F | 1 | 1 | 0 | 8 | 2 | 0 | 10 | 13·2 | 0·33 |
| 3-Methoxy-2-acetamido-fluorene . . . | 35 | 27 | F | 0 | 0 | 0 | 2 | 0 | 0 | 2 | 7·4 | 0·89 |
| *Azocompounds* | | | | | | | | | | | | |
| KB Orange . . | 86 | 62 | F | 0 | 0 | 0 | 31 | 9 | 0 | 40 | 64·5 | <0·001 |
| KB Orange (61 weeks) | — | 9 | F | 0 | 0 | 0 | 4 | 2 | 0 | 6 | 66·7 | } 0·0055 |
| KB Orange (61 weeks) | — | 9 | M | 0 | 0 | 0 | 0 | 0 | 0 | 0 | 0 | } |
| *Others* | | | | | | | | | | | | |
| 20-Methylcholanthrene (0·05 per cent) . . | 78 | 64 | F | 0 | 7 | 0 | 11 | 2 | 0 | 13 | 20·3 | 0·094 |
| 4 mm. glass beads . | 43 | 37 | F | 0 | 1 | 0 | 1 | 2 | 0 | 3 | 8·1 | — |

* Most advanced lesion only counted.

† Quoted by Clayson, *et al.* (1967).

‡ The exact method for 2 × 2 tables was used.

tion in the first experiment with Oil Orange KB, it is apparent from the second experiment that this substance is a potent carcinogen in its own right on bladder implantation.

2, 5-Dimethoxy-1-phenylazo-2-naphthol caused a lower, but still significant, yield of carcinomas on implantation than 1-phenylazo-2-naphthol. The triphenylmethane dyes, Blue VRS and Patent Blue V, were inactive (Table I). The negative result with Blue VRS may be contrasted with the local sarcomas obtained by Walpole (quoted by Grasso and Golberg, 1966) after the subcutaneous injection of an aqueous solution of this dye into the rat. Patent Blue V was inactive on subcutaneous injection in the rat (Truhaut, 1962).

Three aromatic amine derivatives were examined as relevant to the mode of action of their parent amines. N-Hydroxy-2-acetamidofluorene and N-hydroxy-4-acetamidodiphenyl failed to increase significantly the yield of carcinomas over that induced by paraffin wax alone. This is in keeping with the results of Boyland, Busby, Dukes, Grover and Manson (1964) with the latter chemical in cholesterol pellets. They and Bryan *et al.* (1964*b*) obtained a significant yield of carcinomas when N-hydroxy-2-acetamidofluorene was implanted in cholesterol. 1,2-Naphthoquinone-4-sulphonic acid (sodium salt) was investigated because it can combine with amino-groups in protein in a manner similar to the quinone-imines. Its lack of carcinogenicity in this test casts further doubt on the involvement of this type of molecule in aromatic amine carcinogenesis (Nagasawa and Gutmann, 1959).

Neither 1-methoxy- nor 3-methoxy-2-acetamidofluorene induced a significant yield of tumours. As Gutmann, Galitski and Foley (1968) showed that the former compound was carcinogenic on oral administration, this is presumptive evidence that it must be metabolised before it exhibits carcinogenic activity. Commercial *o*-aminoazotoluene, however, induced significantly more tumours than the controls.

The incorporation of 20-methylcholanthrene at a level of 0·5 or 0·05 per cent in paraffin wax failed to increase the incidence of carcinomas to a statistically significant extent compared to the vehicle alone. On the other hand the weak hydrocarbon carcinogen, 1,2-benzanthracene, at a concentration of 12·5 per cent, induced a highly significant yield of carcinomas, none of which had penetrated through the bladder wall.

The use of glass beads (4 mm. diameter) instead of paraffin wax pellets as the implant led to 3 carcinomas in 37 surviving mice (8·1 per cent) in the C57 $\times$ IF mouse. Ball, Field, Roe and Walters (1964) used specially selected smooth and artificially roughened glass beads weighing between 40 and 50 mg. and obtained no carcinomas in 70 mice in the former case and one carcinoma in 67 mice in the latter.

Clayson, Lawson and Pringle (1967) commented on the observation that pellets of paraffin wax alone induced carcinomas in the bladder in female but not in male C57 $\times$ IF $F_1$ hybrid mice. The result was not statistically significant on the number of animals employed. A number of male and female mice of the same hybrid were implanted, for another purpose, with paraffin wax pellets containing Oil Orange KB and were killed 61 weeks later. There were 6 carcinomas in the 9 surviving females but none in the 9 males ($P = 0·006$). The influence of the sex of the mice on the development of bladder tumours has not been extensively investigated.

### DISCUSSION

### *The utility of the technique of bladder implantation*

Clayson (1966) analysed the results of the use of bladder implantation in different centres. Although there were two examples in which apparently similar conditions led to dissimilar results, the technique, otherwise, gave reproducible results. Known locally-active carcinogens, such as the polycyclic aromatic hydrocarbons, were carcinogenic on bladder implantation. Weakly active systemic carcinogens, such as the derivatives of 1-phenylazo-2-naphthol, were more active on bladder implantation than systemically. Oil Orange KB, which was very weakly active on systemic administration to the mouse (Bonser, Clayson and Jull, 1956), was highly active on bladder implantation, giving tumour yields of 98 and 65 per cent in two experiments.

It has been suggested that the results obtained by bladder implantation may be dependent on traces of carcinogenic polycylic hydrocarbons in the paraffin wax of the vehicle. Clayson and Pringle (1966) concluded that, when the vehicle was implanted alone, the induction of bladder epithelial cell turnover was more likely to be important in tumorigenesis than the possible presence of traces of extraneous carcinogens. This is supported by the observation that glass beads induce carcinomas. The results with 20-methylcholanthrene (0·5 per cent) and 1-phenylazo-2-naphthol separately or together indicate that subcarcinogenic amounts of one carcinogen may enhance the tumour yield obtained with another.

The use of bladder implantation for routine testing of carcinogenic activity is complicated by two factors: the stability of the chemical in the pellet, and a lack of information about the transfer of the chemical from the pellet to the bladder epithelium. Irving, Gutmann and Larson (1963) demonstrated that 1-amino-2-naphthol hydrochloride was slowly altered when implanted in paraffin wax pellets into the mouse bladder. The transfer of the chemical from the pellet to the epithelium may occur by direct contact or by the diffusion of the chemical from the pellet into the urine and its subsequent uptake by the bladder. It has been shown (Bryan, Brown, Morris and Price, 1964) that different chemicals diffuse from the pellet at different rates and that certain substances pass easily through the bladder epithelium whether or not a pellet is present, but further information will be required before the behaviour of individual chemicals in these ways can confidently be predicted. Therefore, while a positive result on bladder implantation indicates carcinogenic activity by the chemical, a negative result is inconclusive unless it is demonstrated that the chemical reaches the epithelium. This means that considerable caution must be exercised before a comparison of the carcinogenicity of two or more chemicals is made by bladder implantation. It also offers a partial explanation of the different results obtained with the same chemical in different vehicles. The lack of carcinogenic activity of Blue VRS on bladder implantation may be due to an inability to permeate the bladder epithelium.

It is now apparent that the pellet in the lumen of the bladder induces hyperplasia and mitosis in the epithelium (Clayson and Pringle, 1966). It has also been demonstrated that a bladder pellet enhances the yield of tumours induced by the systemic administration of 4-ethylsulphonylnaphthalene-1-sulphonamide (Clayson and Bonser, 1965), xanthurenic acid-8-methyl ether (Bryan and Springberg, 1966), 2-aminodiphenylene oxide (Clayson *et al.*, 1967) and 2-acetamidofluorene

(Wood, unpublished observation). Thus the technique of bladder implantation detects weak carcinogenic stimuli, a postulate which is supported by the tumour yields obtained with the weakly carcinogenic hydrocarbon, 1,2-benzanthracene, and by the derivatives of 1-phenylazo-2-naphthol.

One of our more interesting results is the finding, albeit in small numbers of animals, that there is a marked sex difference in response to pellets containing Oil Orange KB. Such sex differences have been observed with systemically applied bladder carcinogens but have usually been explained by the suggestion that metabolism may differ between the sexes (Weisburger, Grantham and Weisburger, 1964) or that the animals of one sex have succumbed to tumour formation in other tissues before bladder tumours have had time to develop (Clayson *et al.*, 1967). The present evidence suggests that sex may play a more direct part in the genesis of bladder tumours.

In summary, it is suggested that bladder implantation is a valid method of assessing carcinogenic activity although negative results cannot be accepted without a direct demonstration that the chemical has come into contact with the bladder epithelium. As the pellet stimulates the bladder epithelium, the technique is capable of demonstrating weak carcinogenic activity.

### SUMMARY

1. Pellets of paraffin wax alone induced 5 and 4 per cent carcinomas in separate experiments, while another sample of wax caused 9 per cent carcinomas in female but none in male mice. Glass beads induced 8 per cent carcinomas in female mice.

2. A commercial dyestuff, Oil Orange KB, induced more tumours than any other substance on bladder implantation. Other chemicals found to be carcinogenic were 2,5-dimethoxy-1-phenylazo-2-naphthol, 1,2-benzanthracene and a commercial sample of *o*-aminoazotoluene.

3. N-Hydroxy-2-acetamidofluorene, N-hydroxy-4-acetamidodiphenyl, 1,2-naphthoquinone-4-sulphonic acid (sodium salt), Blue VRS and Patent Blue V did not induce statistically significant yields of tumours.

4. 20-Methylcholanthrene (0·5 and 0·05 per cent) in pellets caused increased but not statistically significant yields of tumours compared to the controls. The former concentration of 20-methylcholanthrene in conjunction with 1-phenylazo-2-naphthol (12·5 per cent) induced more numerous and more advanced tumours than either chemical alone.

5. In a limited experiment it was shown that Oil Orange KB induced carcinomas when implanted into female but not into male mice.

6. The utility of the technique of bladder implantation was discussed in relation to its reproducibility, the significance of negative results and its ability to detect carcinogens of low activity.

We thank the Yorkshire Council of the British Empire Cancer Campaign for Research for financial support.

### REFERENCES

ALLEN, M. J., BOYLAND, E., DUKES, C. E., HORNING, E. S. AND WATSON, J. G.—(1957) *Br. J. Cancer*, **11**, 212.

BALL, J. K., FIELD, W. E. H., ROE, F. J. C. AND WALTERS, M.—(1964) *Br. J. Urol.*, **36**, 225.

BONSER, G. M., BOYLAND, E., BUSBY, E. R., CLAYSON, D. B., GROVER, P. L. AND JULL, J. W.—(1963) *Br. J. Cancer*, **17**, 127.

BONSER, G. M., CLAYSON, D. B. AND JULL, J. W.—(1956) *Br. J. Cancer*, **10**, 653.—(1958) *Br. med. Bull.*, **14**, 146.—(1963) *Br. J. Cancer*, **17**, 235.

BONSER, G. M. AND JULL, J. W.—(1956) *J. Path. Bact.*, **72**, 489.

BOYLAND, E., BUSBY, E. R., DUKES, C. E., GROVER, P. L. AND MANSON, D.—(1964) *Br. J. Cancer*, **18**, 575.

BRYAN, G. T., BROWN, R. R., MORRIS, C. R. AND PRICE, J. M.—(1964) *Cancer Res.*, **24**, 586.

BRYAN, G. T., BROWN, R. R. AND PRICE, J. M.—(1964*a*) *Cancer Res.*, **24**, 582—(1964*b*) *Cancer Res.*, **24**, 596.

BRYAN, G. T., MORRIS, C. R. AND BROWN, R. R.—(1965) *Cancer Res.*, **25**, 1432.

BRYAN, G. T. AND SPRINGBERG, P. D.—(1966) *Cancer Res.*, **26**, 105.

CLAYSON, D. B.—(1966) *Can. Cancer Congr.*, **6**, 186.

CLAYSON, D. B. AND BONSER, G. M.—(1965) *Br. J. Cancer*, **19**, 311.

CLAYSON, D. B., LAWSON, T. A. AND PRINGLE, J. A. S.—(1967) *Br. J. Cancer*, **21**, 755.

CLAYSON, D. B. AND PRINGLE, J. A. S.—(1966) *Br. J. Cancer*, **20**, 564.

GRASSO, P. AND GOLBERG, L.—(1966) *Fd Cosmet. Toxicol.*, **4**, 297.

GUTMANN, H. R., GALITSKI, S. B., AND FOLEY, W. A.—(1968) *Cancer Res.*, **28**, 234.

IRVING, C. C., GUTMANN, H. R. AND LARSON, D. M.—(1963) *Cancer Res.*, **23**, 1782.

JULL, J. W.—(1951) *Br. J. Cancer*, **5**, 328.

NAGASAWA, H. T. AND GUTMANN, H. R.—(1959) *J. biol. Chem.*, **234**, 1593.

PRINGLE, J. A. S.—(1966) *Rep. Br. Emp. Cancer Campn.*, **44**, 255.

TRUHAUT, R.—(1962) *Bull. Soc. scient. Hyg. aliment.*, **50**, 77.

WEISBURGER, E. K., GRANTHAM, P. H. AND WEISBURGER, J. H.—(1964) *Biochemistry, Wash.*, **3**, 808.


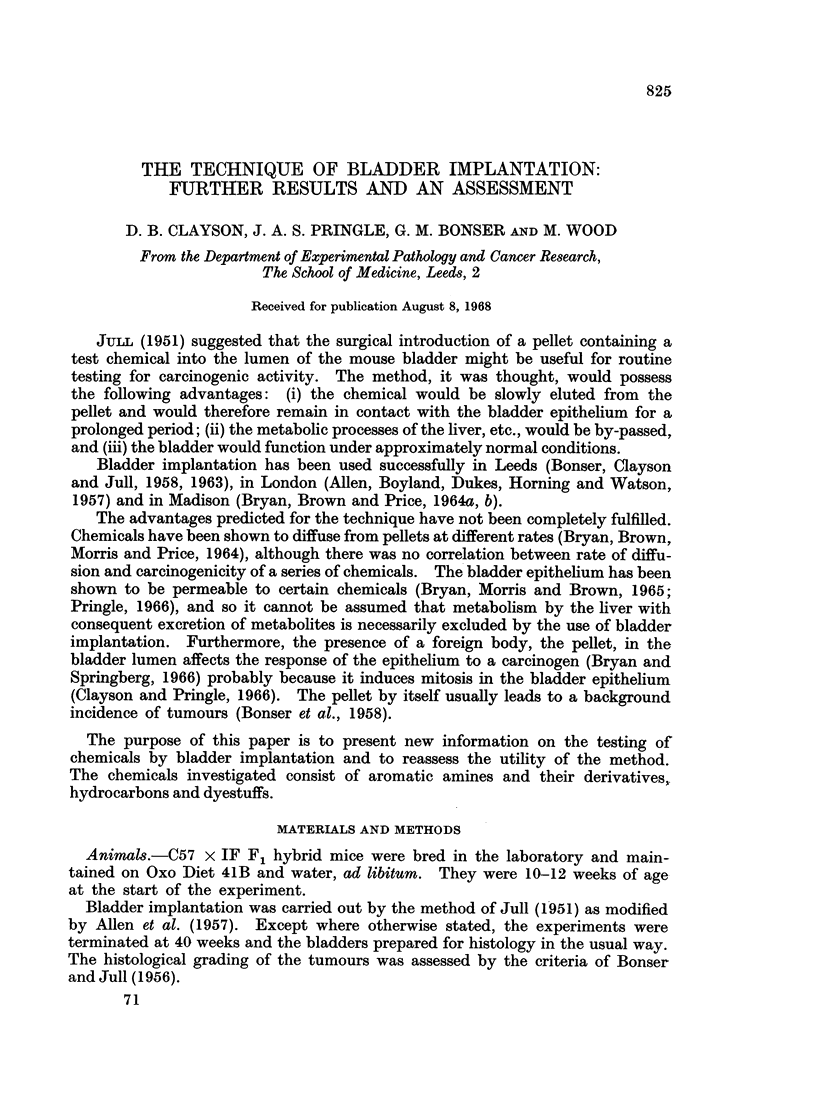

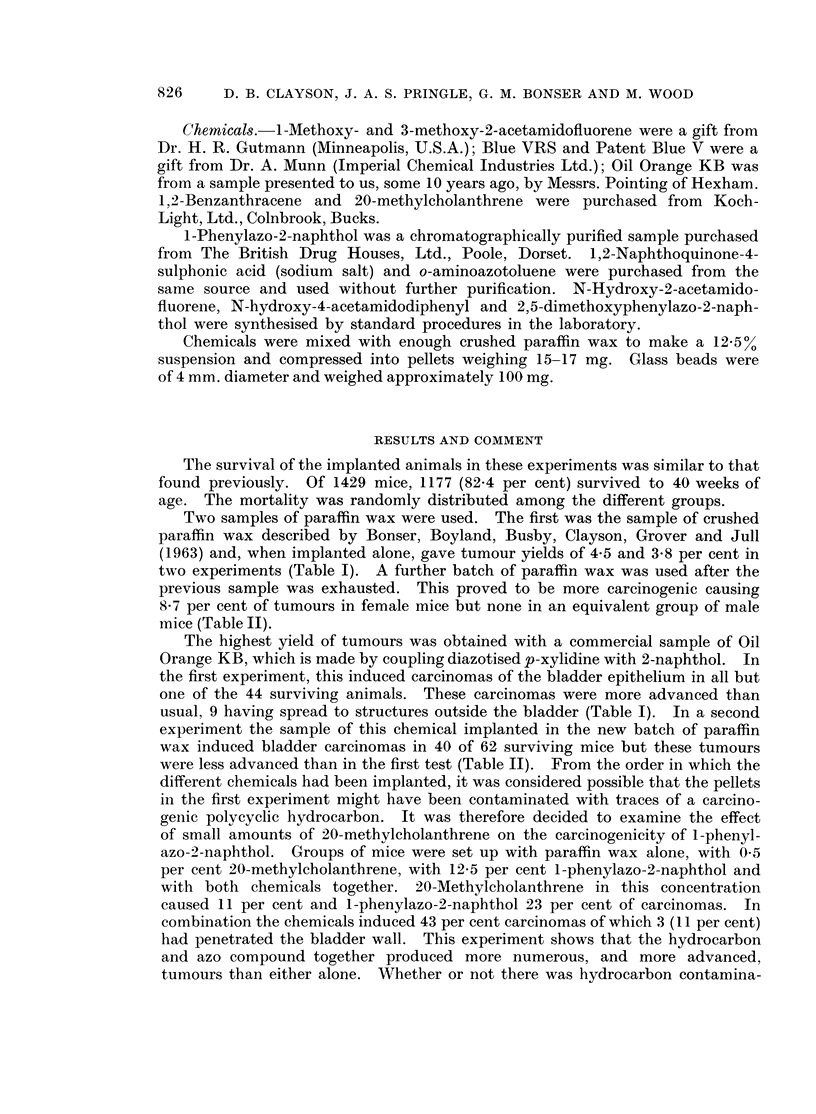

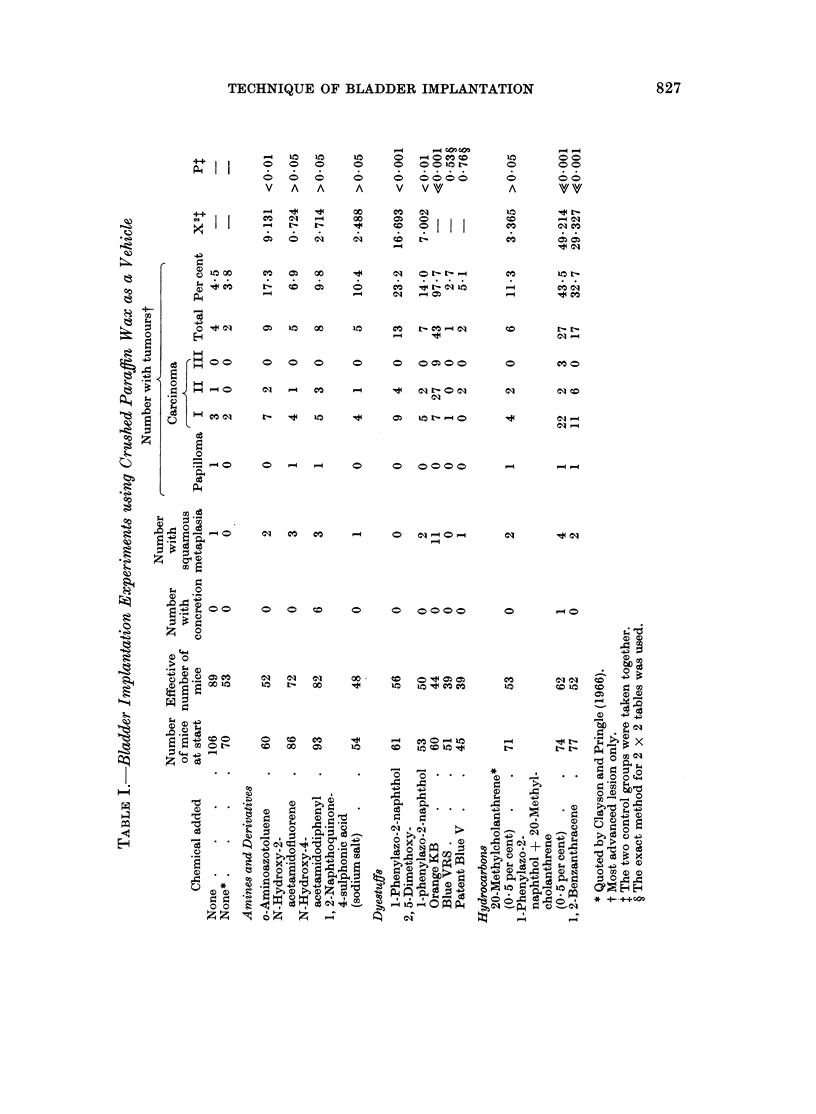

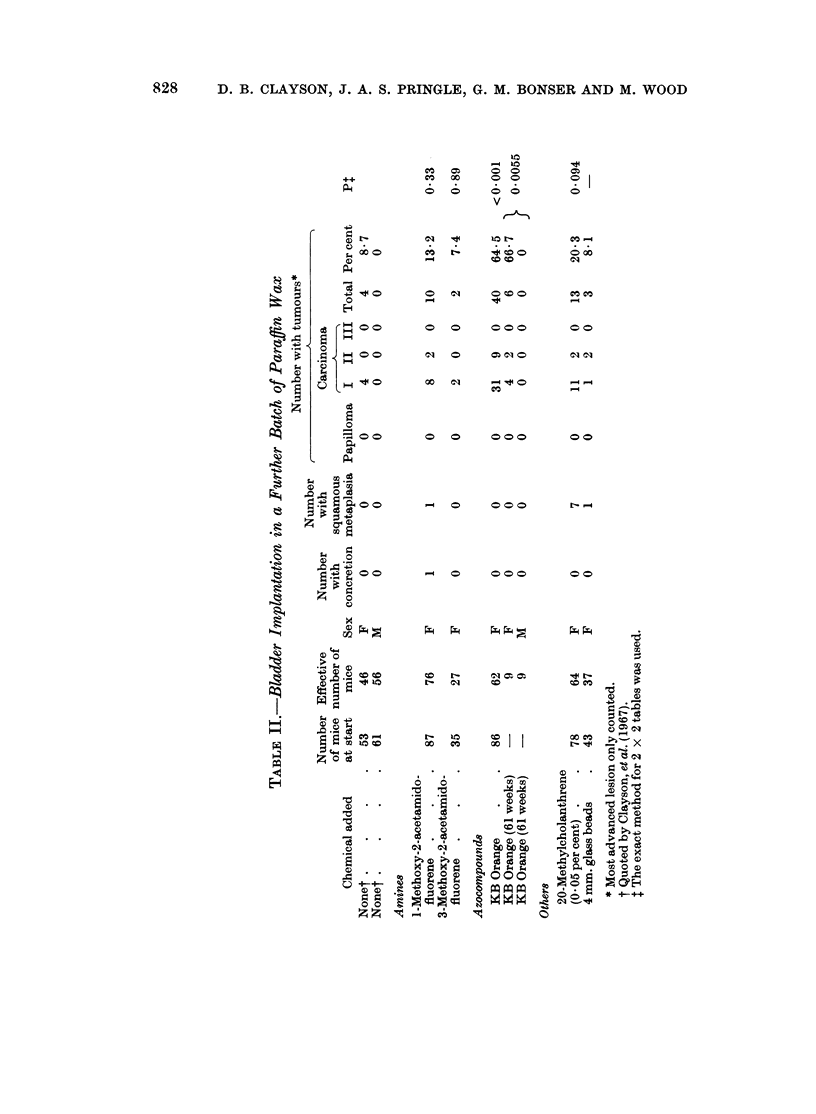

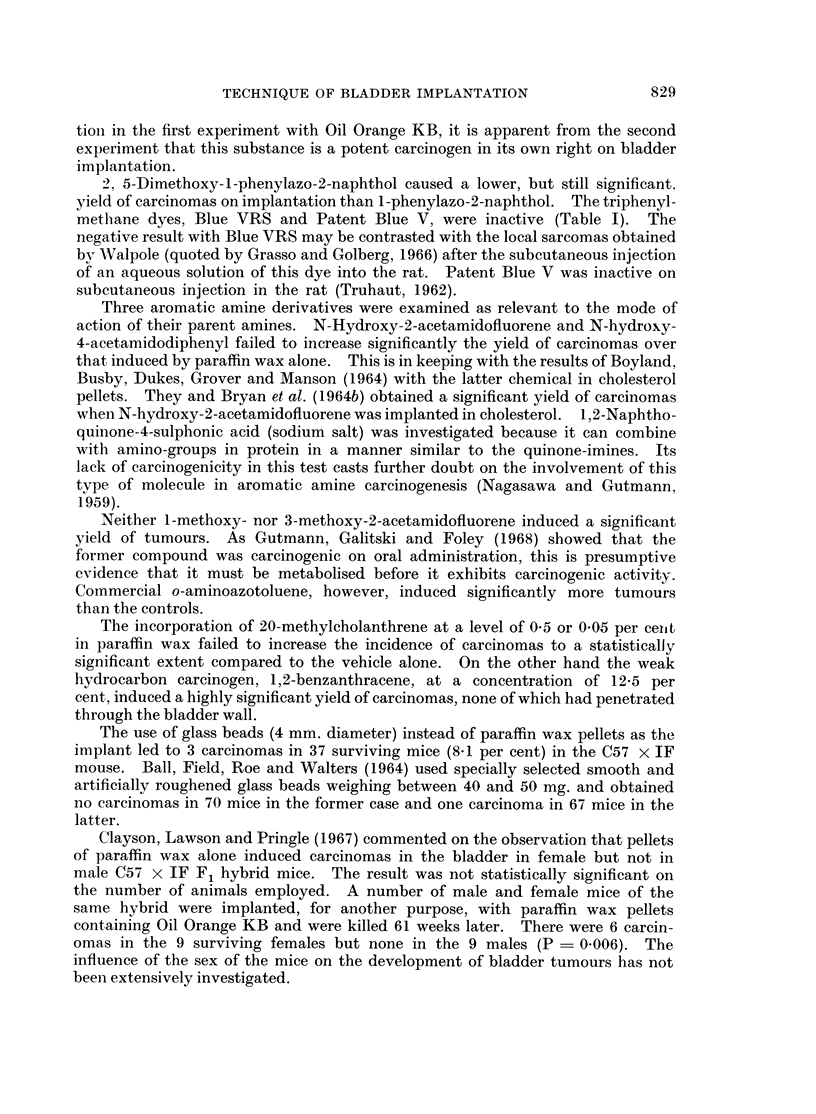

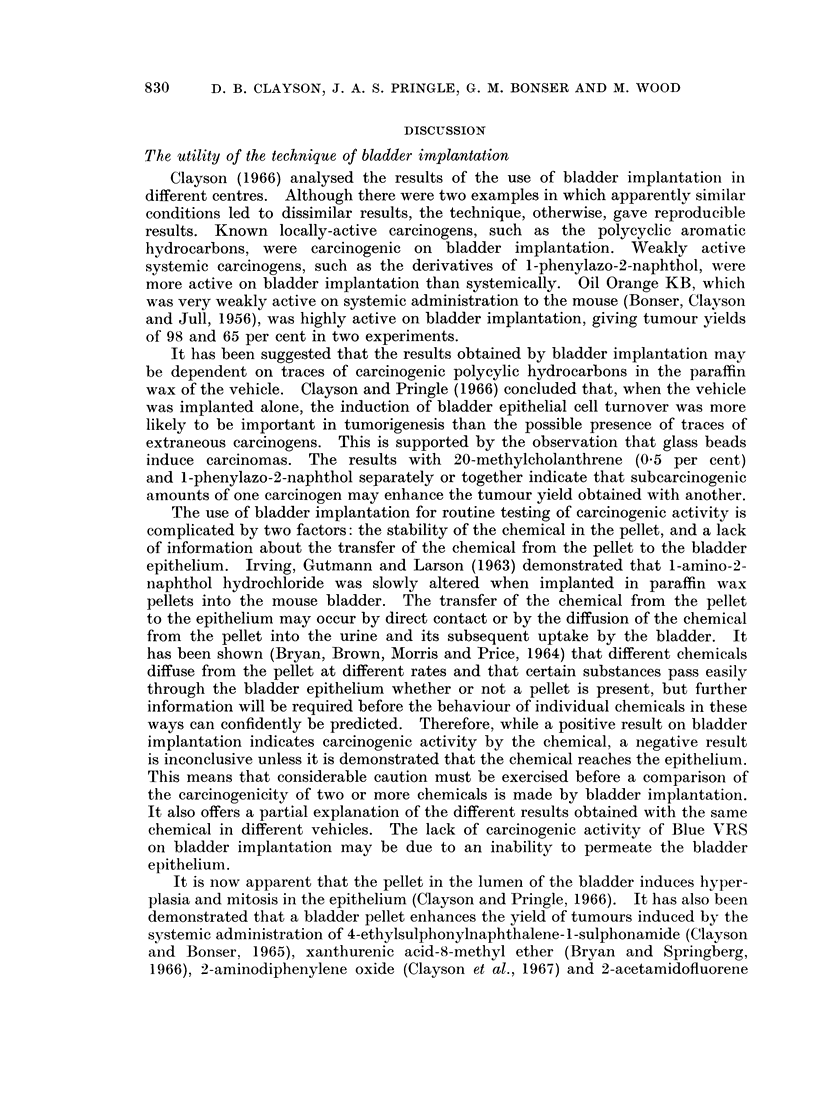

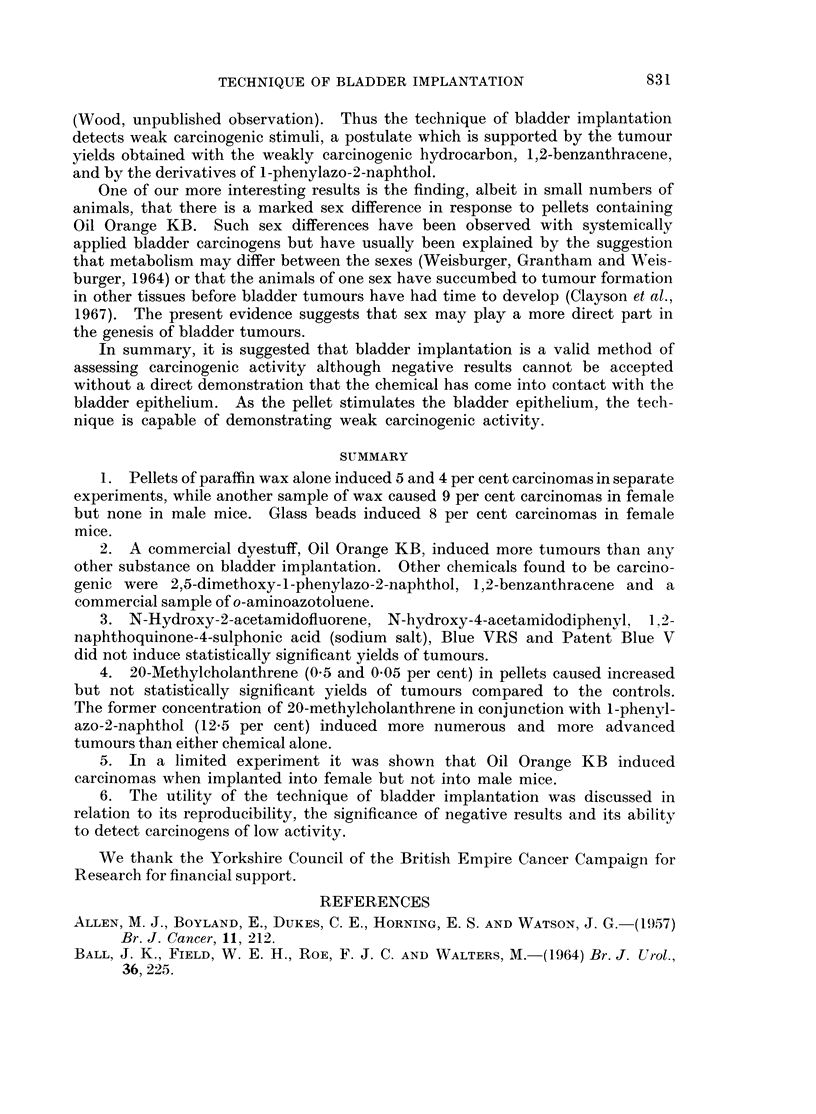

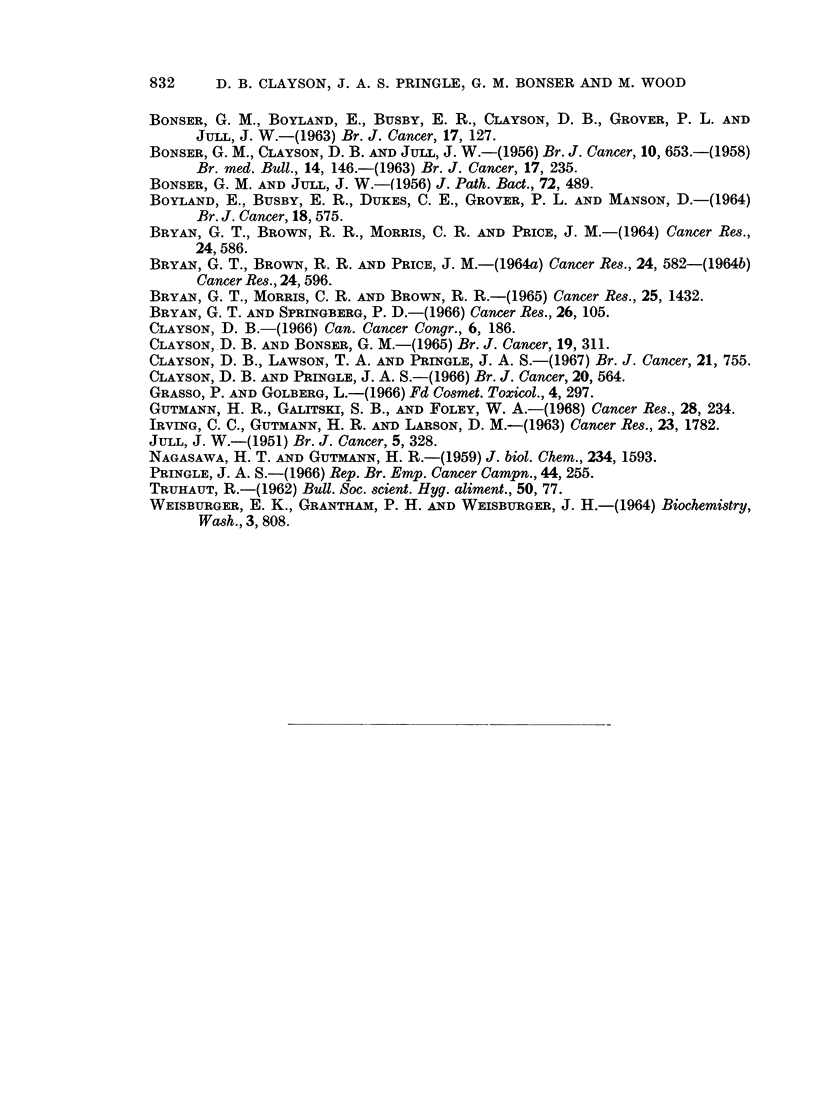


## References

[OCR_00596] ALLEN M. J., BOYLAND E., DUKES C. E., HORNING E. S., WATSON J. G. (1957). Cancer of the urinary bladder induced in mice with metabolites of aromatic amines and tryptophan.. Br J Cancer.

[OCR_00600] BALL J. K., FIELD W. E., ROE F. J., WALTERS M. (1964). THE CARCINOGENIC AND CO-CARCINOGENIC EFFECTS OF PARAFFIN WAX PELLETS AND GLASS BEADS IN THE MOUSE BLADDER.. Br J Urol.

[OCR_00604] BONSER G. M., BOYLAND E., BUSBY E. R., CLAYSON D. B., GROVER P. L., JULL J. W. (1963). A further study of bladder implantation in the mouse as a means of detecting carcinogenic activity: use of crushed paraffin wax or stearic acid as the vehicle.. Br J Cancer.

[OCR_00608] BONSER G. M., CLAYSON D. B., JULL J. W. (1956). The induction of tumours of the subcutaneous tissues, liver and intestine in the mouse by certain dye-stuffs and their intermediates.. Br J Cancer.

[OCR_00616] BOYLAND E., BUSBY E. R., DUKES C. E., GROVER P. L., MANSON D. (1964). FURTHER EXPERIMENTS ON IMPLANTATION OF MATERIALS INTO THE URINARY BLADDER OF MICE.. Br J Cancer.

[OCR_00622] BRYAN G. T., BROWN R. R., MORRIS C. R., PRICE J. M. (1964). IN VIVO ELUTION OF TRYPTOPHAN METABOLITES AND OTHER AROMATIC NITROGEN COMPOUNDS FROM CHOLESTEROL PELLETS IMPLANTED INTO MOUSE BLADDERS.. Cancer Res.

[OCR_00626] BRYAN G. T., BROWN R. R., PRICE J. M. (1964). INCIDENCE OF MOUSE BLADDER TUMORS FOLLOWING IMPLANTATION OF PARAFFIN PELLETS CONTAINING CERTAIN TRYPTOPHAN METABOLITES.. Cancer Res.

[OCR_00632] CLAYSON D. B., BONSER G. M. (1965). THE INDUCTION OF TUMOURS OF THE MOUSE BLADDER EPITHELIUM BY 4-ETHYLSULPHONYLNAPHTHALENE-1-SULPHONAMIDE.. Br J Cancer.

[OCR_00634] Clayson D. B., Lawson T. A., Pringle J. A. (1967). The carcinogenic action of 2-aminodiphenylene oxide and 4-aminodiphenyl on the bladder and liver of the C57 X IF mouse.. Br J Cancer.

[OCR_00630] Clayson D. B. (1966). Metabolites of carcinogens.. Proc Can Cancer Conf.

[OCR_00635] Clayson D. B., Pringle J. A. (1966). The influence of a foreign body on the induction of tumours in the bladder epithelium of the mouse.. Br J Cancer.

[OCR_00636] Grasso P., Golberg L. (1966). Subcutaneous sarcoma as an index of carcinogenic potency.. Food Cosmet Toxicol.

[OCR_00638] Gutmann H. R., Galitski S. B., Foley W. A. (1968). The carcinogenicity of the o-methoxy derivatives of N-2-fluorenylacetamide and of related compounds in the rat.. Cancer Res.

[OCR_00639] IRVING C. C., GUTMANN H. R., LARSON D. M. (1963). EVALUATION OF THE CARCINOGENICITY OF AMINOFLUORENOLS BY IMPLANTATION INTO THE BLADDER OF THE MOUSE.. Cancer Res.

[OCR_00640] JULL J. W. (1951). The induction of tumours of the bladder epithelium in mice by the direct application of a carcinogen.. Br J Cancer.

[OCR_00642] NAGASAWA H. T., GUTMANN H. R. (1959). The oxidation of o-aminophenols by cytochrome c and cytochrome oxidase. I. Enzymatic oxidations and binding of oxidation products to bovine serum albumin.. J Biol Chem.

[OCR_00646] WEISBURGER E. K., GRANTHAM P. H., WEISBURGER J. H. (1964). DIFFERENCES IN THE METABOLISM OF N-HYDROXY-N-2-FLUORENYLACETAMIDE IN MALE AND FEMALE RATS.. Biochemistry.

